# SLAM-OR: Simultaneous Localization, Mapping and Object Recognition Using Video Sensors Data in Open Environments from the Sparse Points Cloud

**DOI:** 10.3390/s21144734

**Published:** 2021-07-11

**Authors:** Patryk Mazurek, Tomasz Hachaj

**Affiliations:** Institute of Computer Science, Pedagogical University of Krakow, 2 Podchorazych Ave, 30-084 Krakow, Poland; patryk.mazurek@up.krakow.pl

**Keywords:** simultaneous localization and mapping, objects recognition, video sensors, deep learning, sparse point cloud, principal component analysis

## Abstract

In this paper, we propose a novel approach that enables simultaneous localization, mapping (SLAM) and objects recognition using visual sensors data in open environments that is capable to work on sparse data point clouds. In the proposed algorithm the ORB-SLAM uses the current and previous monocular visual sensors video frame to determine observer position and to determine a cloud of points that represent objects in the environment, while the deep neural network uses the current frame to detect and recognize objects (OR). In the next step, the sparse point cloud returned from the SLAM algorithm is compared with the area recognized by the OR network. Because each point from the 3D map has its counterpart in the current frame, therefore the filtration of points matching the area recognized by the OR algorithm is performed. The clustering algorithm determines areas in which points are densely distributed in order to detect spatial positions of objects detected by OR. Then by using principal component analysis (PCA)—based heuristic we estimate bounding boxes of detected objects. The image processing pipeline that uses sparse point clouds generated by SLAM in order to determine positions of objects recognized by deep neural network and mentioned PCA heuristic are main novelties of our solution. In contrary to state-of-the-art approaches, our algorithm does not require any additional calculations like generation of dense point clouds for objects positioning, which highly simplifies the task. We have evaluated our research on large benchmark dataset using various state-of-the-art OR architectures (YOLO, MobileNet, RetinaNet) and clustering algorithms (DBSCAN and OPTICS) obtaining promising results. Both our source codes and evaluation data sets are available for download, so our results can be easily reproduced.

## 1. Introduction

Simultaneous localization and mapping (SLAM) is a group of algorithms that serve a purpose of a long-term simultaneous map building and localization with globally referenced position estimation without a priori information [1]. This is a fundament problem in mobile robotics; however, SLAM finds application in many other fields like deep space exploration, indoor localization and navigation in large scenes [2]. In recent years deep neural networks (DNN) has also be applied to enhance the performance of SLAM methods. Most of researchers utilizes DNN to generate image embedding by using convolutional neural network (CNN) based image descriptors [3]. In researches presented in papers [4,5] authors proved that with CNN it is possible to map raw pixels from a single front-facing camera directly to steering commands. It has also been shown that simultaneous application of SLAM and object recognition (OR) system can improve performance of object recognition by supporting OR with additional information about spatial positioning of detected object, also in systems that utilizes monocular visual sensors [6].

### 1.1. The State of the Art

In this subsection we will discuss state-of-the-art researches in the field of SLAM methods, DNN-based objects detection and papers that incorporates both approaches to solve either/or SLAM or OR problems.

#### 1.1.1. SLAM

There are many possible solutions of SLAM problem that depends, i.e., on application and data acquisition sensors that are used to acquire environmental data. The most popular that are used are visual sensors (mono, stereo and multi ocular) [7,8,9], LiDAR [10,11,12,13], RADAR [14], GPS sensors and inertial sensors [15] and others [16]. Among already mentioned technologies visual sensors are popular and affordable devices thanks to the declining price of cameras with sufficiently high resolution and frequency of data acquisition. The monocular ORB-SLAM is considered to be reliable and complete feature-based monocular visual SLAM system [15,17]. It uses Oriented FAST (Features from accelerated segment test) and Rotated BRIEF (Binary Robust Independent Elementary Features) feature detector (ORB) introduced in [18]. In contrary to other stereovision-based solutions [19,20] it requires only a single leans camera which makes it a technically simpler solution with easier calibration procedure. In case of non-visual based solution the most popular approaches use sensors that directly calculates distance between moving object and the environment for example ultrasonic sensor or LiDAR [10,11,12,13]. Application of those measuring devices has some advantages and disadvantages over cameras. Most important advantage is a precision of determining the distance from object and most important disadvantage is sensitivity to interference. Some disadvantages can be corrected by using both solutions in the SLAM algorithm, i.e., distance measurements (LiDAR) and cameras. The results of such solutions are presented in works [21,22]. Additional broad survives on SLAM algorithms can be found in papers [2,17,23,24,25].

#### 1.1.2. Objects Detection Recognition

Application of DNN has revolutionized image-based procedures of features generation, objects detection and recognition [26,27]. This brain-inspired machine learning algorithms [28,29] have found their application, i.e., for security purposes (face detection, vehicle detection) [30,31,32], they are used in autonomous cars to recognize the environment [33], in medicine [34] etc. CNN are robust and relatively fast architectures that with an aid of GPU-based acceleration can be trained to serve in various images domains. Most popular objects detection and recognition models such as R-CNN [35], SPP-NET [36], Fast-RCNN [37], SSD [38], RetinaNet [39], and YOLO [40] have ability to recognition objects in real time what makes them applicable in many practical real life (not only experimental) scenarios. In contrary to pure-classification (objects recognition) architectures like VGG16 [41], InceptionV3 [42] or ResNet [43] objects detection and recognition models are capable to also detect (indicate position in raster images) of many objects simultaneously in real time. The newest versions of YOLO network (namely YOLO v3 and YOLO v4 [44]) seems to be very promising approaches which over perform other state of the art solutions both in detection quality and processing speed.

#### 1.1.3. Localization, Mapping and Objects Recognition

SLAM can also be used to enhance recognition capability of image detection and classification algorithms. In [6], authors propose multi-view object recognition algorithm that uses SLAM for small interior environment mapping by applying dense SITF and maximum-likelihood estimate classifier for objects recognition. Paper [45] proposes an idea of mapping methodology for large-scale outdoor scenes in autonomous off-road driving applications. The semantic map representation consists of a large-scale topological map built using semantic image information by application of SLAM and segmentation algorithm. The application of CNN to enhance the capability or performance of SLAM methods has been already proposed in several researches. In [3], authors apply DNN for image feature extractions. They evaluates DNN pre-trained on ImageNet [46] and Places dataset [47]. Similar approach has been proposed in [48]. A survey on such application of DNN in SLAM can be found in [49,50]. The authors of [51] propose a Semantic SLAM system which builds the semantic maps with object-level entities, and it is integrated into the RGB-D (color and depth channel) SLAM framework. After getting key frames from ORB-SLAM YOLO is used to detect objects in each key frame. Authors have used tiny-weight version to detect objects which is trained on MS-COCO Dataset. In order to find correspondence between existing objects and temporary objects authors used Kd-Tree. The similar approaches that incorporates RGB-D sensor, SLAM and YOLO is proposed in [52]. Paper presents approach to develop a method for an unmanned ground vehicle to perform inspection tasks in nuclear environments using rich information maps. A DNN (namely Tiny YOLO) performs detection of object of a single class—cylinder shaped containers.

#### 1.1.4. Point Clouds Clustering

ORB-SLAM algorithms in order to determine the position of the moving object calculates spatial coordinates of common points that are present in neighbour key frames. Those points, if they belong to the same object, might be then used to determine spatial position of that object. We can assume that ORB-SLAM generates a sparse point cloud which contains points that might belong to several objects that are present in the 2D image. In order to assign those points to certain objects we need to perform clustering of that point cloud which is quite typical approach [53,54,55]. Depending of the cluster characterization one can apply either centroid-based (i.e., k-maxoids [56], k-medoids [57]) or density-based (i.e., FDP [58], DBSCAN [59]) approaches.

### 1.2. Study Motivation

As can be seen from state-of-the-art survey, combining SLAM and OR methods in order to mutually enhance their performance is and open and not trivial task. In this paper, we propose a novel approach that enables simultaneous localization, mapping and objects recognition using visual sensors data in open environments that is capable to work on sparse data point clouds. Our approach is most similar to [51,52]; however, we do not use RGB-D sensors, rather monocular RGB sensor. In the proposed algorithm the ORB-SLAM uses the current and previous monocular visual sensors video frame to determine observer position and to determine a cloud of points that represent objects in the environment, while the deep neural network uses the current frame to detect and recognize objects. In the next step, the sparse point cloud returned from the SLAM algorithm is compared with the area recognized by the OR network. The clustering algorithm determines areas in which points are densely distributed in order to detect spatial positions of objects. Next, the principal component analysis (PCA) based heuristic estimates bounding boxes of detected objects. The image processing pipeline that uses sparse point clouds generated by SLAM in order to determine positions of objects recognized by deep neural network and mentioned PCA heuristic are main novelties of our solution. In contrary to state-of-the-art approaches, our algorithm does not require any additional calculations like generation of dense point clouds for objects positioning, which highly simplifies the task. We have evaluated our research on large benchmark dataset using various state-of-the-art OR architectures (YOLO, MobileNet, RetinaNet) and clustering algorithms (DBSCAN and OPTICS).

### 1.3. Comparison with Other OR-Based Location Recognition Algorithms

Algorithms that perform the task of locating and determining the orientation of objects acquired by a single-lens camera are designed to perform only these actions. This means that these algorithms do not directly solve the SLAM problem but only perform the spatial localization and orientation determination of the object relative to the observer (vehicle) position. These OR algorithms to be used in SLAM and OR tasks simultaneously must be combined with some SLAM class algorithm. This combination results in duplication of computation of similar tasks, since various SLAM algorithms (for example, ORB-SLAM) already allow the determination of spatial coordinates of points that are part of the environment.

Since object detection algorithms that do not use SLAM results must perform simultaneous spatial localization and orientation of objects, solutions that are proposed in the literature create much more complex deep architectures than are required in practice to solve the simultaneous SLAM and OR problem. In practice, in recent studies, authors use a variety of deep architectures to perform simultaneous detection and spatial orientation of objects. These approaches use the generation of pseudo-LiDAR data [60], generating pseudo-depth data [61,62] or explore various 3D structure information of the object by employing the visual features of visible surfaces and 2D to 3D reprojection [63,64,65,66,67,68]. The algorithm proposed in our work, by using data from SLAM has the ability to use general purpose DNNs, which are easier to train due to the availability of multiple training datasets and have a larger capacity for different classes of objects. Algorithms that are present in the literature that perform the OR and localization task allow us usually to work with one class of objects at a time (e.g., only with cars, cyclists, etc.). All these facts make the SLAM-OR algorithm proposed in this work a new and useful approach that can be used when we need to perform both SLAM and OR task simultaneously.

## 2. Material and Methods

In this section, we will explain algorithms that are used in our approach and we will present data sets we have used for training and validation.

### 2.1. Simultaneous Localization and Mapping

Our image processing pipeline incorporates the monocular ORB-SLAM algorithm [69]. We will validate this choice later by comparing its capabilities with other state-of-the-art approaches. That algorithm is divided into three parts: tracking, local mapping and loop closing. We have to remember that in order to work correctly the camera sensors have to be calibrated [70]. As a result of correct calibration reduction of generic distortions is achieved and algorithm obtains more accurate results.

#### 2.1.1. Tracking

The first step of the algorithm is to extracting features from the image, for the purpose of using the ORB algorithm. Features are extracted using FAST [71] and BRIEF [72] algorithms. Images are converted to grayscale, and then FAST algorithm is searching for corners. FAST consists of detecting set of pixels N from the image within a certain radius that meet the threshold value. The ORB algorithm is using the intensity centroid method [73] and is able to determine the orientation of a corner. Assuming that the intensity is shifted from its centre, the resulting vector will help to determine the orientation. The main idea of BRIEF algorithm is to pick random points around the feature points (corner), then combine the value of the points in the binary vector and treat the vector as a descriptor. Equation (1) represent a binary vector T, where g assumes image smoothing

(1) $$T\left(g;x,y\right)=\left\{\begin{array}{c} 1;g(x)<g(y)\\ 0;g(x)\geq g(y) \end{array}\right.$$

where g(x) id is defined as (2)

(2) $$f_{n}\left(g\right)=\sum_{1\leq i\leq n}2^{i-1}T\left(g;x_{i},y_{i}\right)$$

The ORB extracts the BRIEF descriptors according to the direction obtained by Equation (3):

(3) S=x1,...,xny1,...,yn

We can construct $S_{\Theta }$ from S using the appropriate rotation matrix $R_{\Theta }$ and orientation Θ (9)

(4) $$S_{\Theta }=R_{\theta }S$$

Returned descriptors are sensitive to interference and noise, which might cause a large variability of each feature. ORB dynamically adjusts the threshold value for the descriptors and selects ones with the highest value. In the situation when there are no features in certain parts of the images (for example due to a uniform background or low difference in contrast) the ORB uses other descriptors. If there are not enough features to perform tracking the search for points is repeated based on the current position. After obtaining the appropriate feature from the image and using the DBoW2 mechanisms [74], the predicted position of the camera is determined.

#### 2.1.2. Mapping

Once the features’ positions are estimated, a local map using BA [75] is derived. Then new equivalents of mismatched ORB points are searched from the remaining key frames to triangulate the new points. The algorithm monitors the resulting points and, on the basis of the information collected during the tracking, it eliminates the points so that only the highest quality points remain.

#### 2.1.3. Loop Closing

The last step of ORB-SLAM is to compare the obtained local map with the last key frames in order to optimize the duplicate points that will be merged. After that, ORB-SLAM proceeds to the search for the next key frame.

### 2.2. YOLO

YOLO (You Only Look Once) [40] is a neural network-based approach to recognize objects. The image that is subjected to the detection process at the beginning is divided into a grid with dimensions S∗S. After dividing the image, each sector is in the same time subjected to recognition and the bounding boxes B are defined if the certain object has been detected within the frame. Bounding box stores following information: $c_{x},c_{y}$—position of centre of the object, w, h—dimensions of the object, Con—probability of the presence of the object in the frames. Probability is defined as:

(5) $$Con=Pr\left(object\right)*IOU_{predict}^{truth}$$

In Equation (5) IOU is represented as a value in the range from 0 to 1, and it is the sum of the intersection of the predicted frame with the bases truth. If the object is not in the mesh, then it gets the value 0. Then the classes probability $Pr(class_{i}|object_{i})$ (6) is determined for each mesh.

(6) $$Pr\left(class_{i}|object\right)*Pr\left(object\right)*IOU_{predict}^{truth}=Pr\left(class_{i}\right)*IOU_{predict}^{truth}$$

Only one class probability is predicted in each grid, regardless of the number of bounded boxes. As a result, we get the output tensor S∗S∗(B∗5+Con). Then the centre and frame position is corrected by the loss function (7).

(7) $$\begin{array}{c} Loss=\lambda_{coord}\sum_{i=0}^{S^{2}}\sum_{j=0}^{B}I_{ij}^{obj}\left[{\left(c_{x_{i}}-{\hat{c}}_{x_{i}}\right)}^{2}+{\left(c_{y_{i}}-{\hat{c}}_{y{}_{i}}\right)}^{2}\right]\\ +\lambda_{coord}\sum_{i=0}^{S^{2}}\sum_{j=0}^{B}I_{ij}^{obj}\left[{\left(\sqrt{w_{i}}-\sqrt{{\hat{w}}_{i}}\right)}^{2}+{\left(\sqrt{h_{i}}-\sqrt{{\hat{h}}_{i}}\right)}^{2}\right]+\sum_{i=0}^{S^{2}}\sum_{j=0}^{B}I_{ij}^{obj}{\left(Con_{i}-{C\hat{o}n}_{i}\right)}^{2}\\ +\lambda_{noobj}\sum_{i=0}^{S^{2}}\sum_{j=0}^{B}I_{ij}^{noobj}{\left(Con_{i}-{C\hat{o}n}_{i}\right)}^{2}+\sum_{i=0}^{S^{2}}I_{ij}^{obj}\sum_{Con\epsilon classes}{\left(p_{i}\left(Con\right)-{\hat{p}}_{i}\left(Con\right)\right)}^{2} \end{array}$$

where B are bounding boxes in each grid, $\lambda_{coord}$ and $\lambda_{noobj}$ are used to increase the emphasis on a grid. $p_{i}\left(Con\right)$ references to the classification prediction, value $I_{ij}^{obj}$ is in the range 0 to 1 depending the probability of the object in the j-th bounding box and i-th cell. The $I_{i}^{obj}$ is 1 if there is an object in the i-th cell, or 0 in not. YOLO achieves high real-time performance, but each field of the mesh is responsible for one class only, so when there are several centres of objects in one mesh, only the one with the highest IOU is selected as the output. It might be difficult to recognize any overlapping or tiny objects. YOLO is among the fastest and most accurate deep objects detectors and it has several variants, most notably: YOLO V3 [76], YOLO v4 [44] and their tiny (or light) versions [77,78].

### 2.3. RetinaNet

Another efficient and effective objects detection and recognition algorithm is RetinaNet [39]. Authors of that deep neural network address issue of the extreme foreground-background class imbalance encountered during training by reshaping the standard cross entropy loss such that it down-weights the loss assigned to well-classified examples by adding so called Focal Loss factor ${\left(1-p_{t}\right)}^{\gamma }$; where $p_{t}$ is the model’s estimated probability for the certain class. Setting γ>0 reduces the relative loss for well-classified examples putting more focus on hard, misclassified examples. RetinaNet is a single, unified network composed of a backbone network, namely ResNet [43] with Feature Pyramid Network [79] and two task specific subnetworks: for classifying anchor boxes and for regressing from anchor boxes to ground-truth object boxes.

### 2.4. MobileNet

According to [80] depthwise convolution is very efficient relative to standard convolution; however, it only filters input channels and does not combine them to create new features. Due to this fact authors have added combination of depthwise convolution and 1 × 1 (pointwise) convolution called depthwise separable convolution. The MobileNet structure is built on depthwise separable convolutions except for the first layer which is a full convolution. All layers are followed by a batchnorm [81] and ReLU nonlinearity except for the final fully connected layer with softmax activation.

### 2.5. DBSCAN

DBSCAN clustering algorithm operates on the basis of neighbourhood density. The algorithm has $O(n^{2})$ computational complexity and is capable of matching points to sets of irregular shapes. The DBSCAN works as follows: at the beginning, the point p is selected from the set D and the radius $M_{ps}$ (eps) is determined. In the next step it finds points q that meet constraint (8):

(8) $$M_{ps}\left(p\right)=\left\{q\epsilon D|dist\left(p,q\right)\leq M_{ps}\right\}$$

Points that match the condition (8) in order to belong to the cluster must reach a certain density which is defined as $min_{p}$ (min samples) and they satisfy two conditions (9) and (10):

(9) $$q\epsilon M_{ps}\left(p\right)$$

(10) $$\left|M_{ps}\left(p\right)\right|\geq min_{p}$$

If the condition (10) is satisfied then the point p can be called the focal point from which the cluster is determined. The q farthest points are called boundary points.

The algorithm works until all points are assigned to clusters, and in the case when points are not assigned to clusters $C_{1},\ldots ,C_{m}$, remain in the set D, these points are considered noise and satisfy condition (11)

(11) $$p_{noise}=\left\{q\epsilon D|\forall i:q\notin C_{i}\right\},i=1,...,m$$

### 2.6. OPTICS

OPTICS algorithm [82] is an extension of DBSCAN clustering. It creates a density-based cluster ordering structure of variable neighborhood radius. In order to automatically extract appropriate sets of parameters that indicates clusters an additional reachability data is extracted [83].

### 2.7. SLAM-OR Algorithm

**Figure 1:**
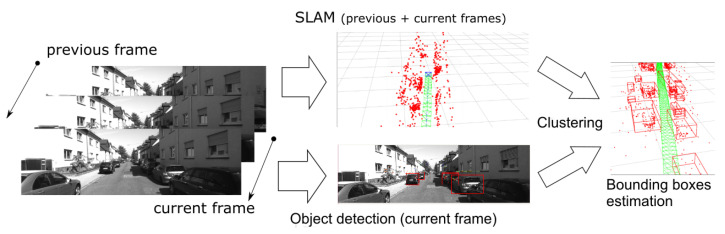
This figure presents the basic concept of the SLAM-OR algorithm.

In the SLAM-OR algorithm, we have used techniques discussed in previous section. Figure 1 presents the basic concept of the SLAM-OR algorithm. The ORB-SLAM algorithm uses the current and previous frames to determine the observer position and to determine a cloud of points that represent all tracking features in the environment. Object detection and recognition DNN uses the current frame to detect and recognize objects. In the next step, the sparse point cloud returned from the SLAM algorithm is compared with the area recognized by the DNN network. Because each point from the 3D map has its counterpart in the current frame, therefore the filtration of points matching the area recognized by the DNN is performed.

Next step of SLAM-OR is point cloud clustering. The aim of this is to aggregate neighbour points in cloud, possible obtained from several following video frames in order to detect all points that describes the same object. After that aggregation we can estimate the spatial coordinates of bounding box of the detected object. Because we cannot make any assumptions about number of clusters that are present in the recording and because cluster membership its determined by spatial closeness and points density the straightforward choice is application of density-based clustering like DBSCAN or, i.e., OPTICS.

The final step of SLAM-OR is estimation of object’s bounding box. We propose a principal components analysis (PCA)—based heuristic. Let us assume that bounding boxes are rectangular. Let us consider all points that are assigned to the cluster as the data source for PCA. After normalization, we use those data to calculate covariance matrix and we calculate eigen decomposition of it, obtaining (in two dimensional case) two eigenvector-eigenvalue pairs $\left\{\left(\bar{v_{1}},\lambda_{1}\right),\left(\bar{v_{2}},\lambda_{2}\right)\right\}$. In the two-dimensional case (which can be easily expanded to three-dimensions), we can assume that first principal component is parallel to two borders of the bounding box ($\bar{b_{1}},\bar{b_{3}}$), while the second principal component is parallel to two remaining bounding box borders ($\bar{b_{2}},\bar{b_{4}}$):

(12) $$\bar{b_{1}}\left|\right|\bar{b_{3}}\left|\right|\bar{v_{1}};\bar{b_{2}}\left|\right|\bar{b_{4}}\left|\right|\bar{v_{2}};\bar{v_{1}}⊥\bar{v2}$$

The centre of the bounding box is situated in the point determined by mean vale of all points in the given cluster *M*, and length of bound box borders is somehow proportional to variance explained by a given principal components that is $var_{\%1}$ for $\bar{b_{1}}$ and $\bar{b_{3}}$; $var_{\%2}$ for $\bar{b_{2}}$ and $\bar{b_{4}}$. Bounding box edges can be computed using following equations:

(13) $$\left\{\begin{array}{c} \bar{b_{1}}=\left[\bar{m}-\bar{l_{1}}-\bar{l_{2}};\bar{m}+\bar{l_{1}}-\bar{l_{2}}\right]\\ \bar{b_{3}}=\left[\bar{m}-\bar{l_{1}}+\bar{l_{2}};\bar{m}+\bar{l_{1}}+\bar{l_{2}}\right]\\ \bar{b_{2}}=\left[\bar{m}-\bar{l_{1}}-\bar{l_{2}};\bar{m}-\bar{l_{1}}+\bar{l_{2}}\right]\\ \bar{b_{4}}=\left[\bar{m}+\bar{l_{1}}-\bar{l_{2}};\bar{m}+\bar{l_{1}}+\bar{l_{2}}\right] \end{array}\right.$$

where:

(14) $$\left\{\begin{array}{c} \bar{l_{1}}=\bar{v_{1}}*\alpha *\sqrt{var_{\%1}}\\ \bar{l_{2}}=\bar{v_{2}}*\alpha *\sqrt{var_{\%2}} \end{array}\right.$$

**Figure 2:**
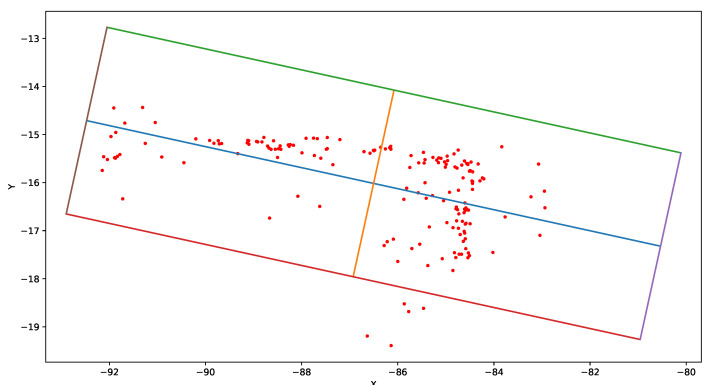
This figure presents example results of objects bounding box calculation using PCA—based approach from Equation (13). Values on the X and Y axis are points coordinates calculated by our algorithm. The scaling of the axes is irrelevant because PCA normalizes variables. Red points are directly calculated from SLAM algorithm. Blue section lays along direction of the highest variance of red points (the first principal component $\bar{v_{1}}$). Orange section is perpendicular to blue section and it lies along direction of the second highest variance of red points (the second principal component $\bar{v_{2}}$). The bounding box have four edges: violet ($\bar{b_{4}}$), green ($\bar{b_{3}}$), brown ($\bar{b_{2}}$) and red ($\bar{b_{1}}$).

α is a scaling parameter, which we authoritatively set to 2. Figure 2 visualizes the example bounding box designated according to the proposed method.

### 2.8. Data Set

In this research we have used two main datasets. For the training of OR network image recognition model we have utilized 5000 images from the OIDv4 database https://github.com/EscVM/OIDv4_ToolKit, accesssed on 10 July 2021. The models were trained to recognize selected objects: cars, bicycles, vans, trucks, buses, persons, trams, traffic lights and traffic signs. For the ORB-SLAM algorithm verification and for evaluation of the whole SLAM-OR framework, we have used a part of the Kitti database [84]. Details about the experimental setup are presented in the following Section 3.

## 3. Results

We have performed several evaluations. At first we wanted to verify if ORB SLAM has the best performance over mono ocular algorithms, with available source codes, on the open environment benchmark. Next, we have evaluated deep objects recognition algorithms. Then, we have performed evaluation of proposed SLAM-OR approach in order to generate a bird’s eye view map of a route followed by the vehicle and the positioning of neighbouring objects. We have tested various OR networks and parameters of clustering algorithms in order to find the best setup for our needs. Most calculations were performed on an Intel Core i5-5200U CPU, 2.20 GHz, 12 GB RAM memory and NVidia 830 M graphics card on Ubuntu 18.04 operating system. Evaluation of the result was performed on an Intel Core i7-9700F CPU, 3 GHz, 64 GB RAM on a Windows 10 operating system. Teaching neural network was performed using the Google Collaborator service. We have implemented our algorithm in Python 3. We have used SciPy implementations of DBSCAN and OPTICS algorithms.

### 3.1. SLAM Algorithm Validation

In order to select the best available visual-based SLAM solution for our needs we have adapted methodology of Kitti odometry benchmark available at http://www.cvlibs.net/datasets/kitti/eval_odometry.php, accessed on 10 July 2021, and compared it with results obtained by us with ORB SLAM [69] using Kitti raw data sets: 09, 22, 23, 35, 39, 46, 61, 64, 84 and 91. We have selected those particular raw data sets because they also contain reference information about objects present in the recordings, which will be needed later. We have taken into account only published algorithms with source codes available. As we can see in Table 1, ORB SLAM outperforms the best single camera algorithm; however, as expected, it has worse results than stereo and LiDAR-based algorithms. The ORB-SLAM results are sufficient for our needs and we decided to apply it for further research. The rest of our evaluation incorporates this algorithm.

### 3.2. Selection of Object Recognition Algorithm

**Figure 3:**
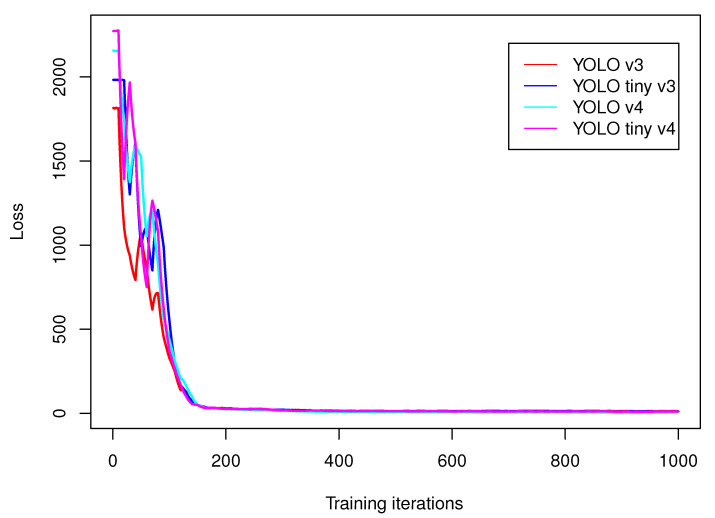
This figure presents plots of loss function (7) values that changes over training on subset of OIDv4 dataset.

We have trained YOLO v3, YOLO v3 tiny, YOLO v4, YOLO v4 tiny, MobileNet and RetinaNet on OIDv4 data set subset introduced in Section 2.8. The training setup was: 1000 iterations, batch size: 64, momentum: 0.949, decay: 0.0005. We have taken 4000 elements to training and 1000 to test data set. Figure 3 presents plots of loss function (7) values of YOLO algorithms that change over training. After approximately 180 iterations the decreases of loss function stabilizes in the long plateau that remains nearly unchanged till the end of the training. The final value of loss function for those networks is presented in Table 2; calculations have been done on an Intel Core i5 PC. The best results regarding loss have been obtained for YOLO. We have also validated data processing frequency (number of frames per second processed by each algorithm—fps) and mean averaged precision (mAP). In case of fps, the fastest network was YOLO v4 tiny, while the highest mAP was obtained by MobileNet. We will perform evaluation of SLAM-OR algorithms on YOLO v4, MobileNet and RetinaNet because those three networks architectures have obtained highest mAP values.

### 3.3. SLAM-OR Evaluation

The last experiment was the evaluation of the capability of a robust bird’s eye view map generation of a route followed by the vehicle and positioning of neighbouring objects. The route and the camera position are direct outputs of the SLAM algorithm. We generated a bird’s eye view map using 10 RAW data sets of the Kitti dataset incorporating information about tracklets (bounding boxes of objects that are visible on recordings) namely 09, 22, 23, 35, 39, 46, 61, 64, 84 and 91 datasets. We have projected all tracklets on horizontal plane in order to create a reference dataset. Then we have applied the SLAM-OR approach to generate vehicle track, perform objects recognition and mapping them on the bird’s eye view map. Then we have compared it to reference data. We have examined following range of parameters of DBSCAN algorithm: eps = {0.5, 0.75, 1.0, 1.25, 1.5}, min samples = {3, 5, 7} and following range parameters of OPTICS algorithm min samples = {5, 10, 15, 20, 25}. Both reference and SLAM-OR maps have been rendered to the raster binary images with resolution 1700 × 700 pixels, $Ref=\{R_{1},...,R_{10}\}$ and $SLAM-OR_{eps,min}=\left\{S_{1},...,S_{10}\right\}$. Bounding boxes interiors have been labelled with bit value 1 while background with 0. In order to judge the quality of the final solution we have calculated following parameters: true positive rate (TPR), positive predictive value (PPV), false negative rate (FNR), F1 score (F1) and Fowlkes–Mallows index (FM). They are defined as follows:

(15) $$TPR=\frac{TP}{TP+FP}=1-FNR$$

(16) $$PPV=\frac{TP}{TP+FP}$$

(17) $$F1=\frac{2\cdot TP}{2\cdot TP+FP+FN}$$

(18) $$FM=\sqrt{PPV\cdot TPR}$$

where TP for pair $R_{i}$ and $S_{i}$ is a number of pixels that have value 1 both in $R_{i}$ and $S_{i}$, FP is number of pixels that have value 1 in $S_{i}$ while they have value 0 in $R_{i}$, FN is number of pixels that have value 0 in $S_{i}$ while they have value 1 in $R_{i}$.

Because results from OPTICS clustering performed on point cloud from SLAM-OR with YOLO v4 network (see Table 3) turned out to be worse than results from DBSCAN clustering performed on SLAM-OR with YOLO v4 (see Table 4), we later used only DBSCAN clustering. Evaluation results for SLAM-OR utilizing DBSCAN with MobileNet and SLAM-OR utilizing DBSCAN with RetinaNet are presented in Table 5 and Table 6. Results in Table 3, Table 4, Table 5 and Table 6 are averaged over all ten Kitti data sets and presented as average value plus/minus standard deviation.

**Figure 4:**
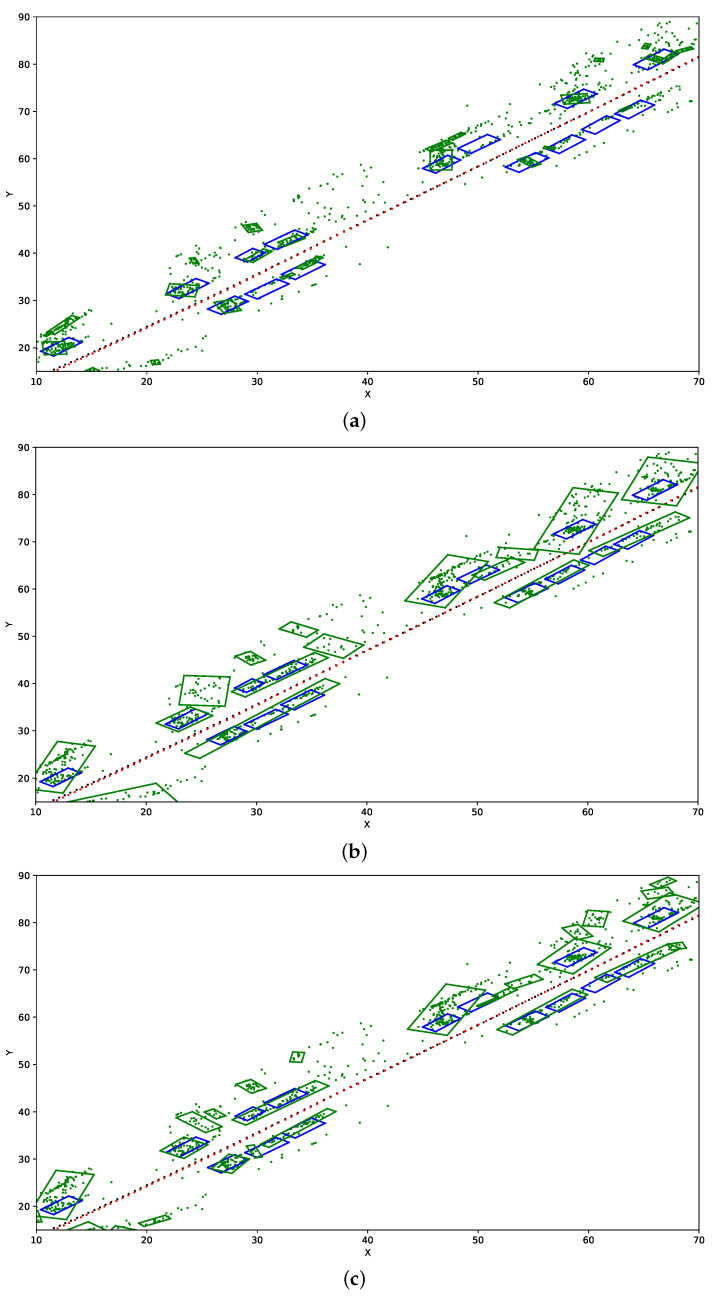
This figure presents influence of application of various DBSCAN parameters on detected objects bounding boxes. Visualization has been done on fragment of the Kitti 39 data set. (**a**) DBSCAN eps = 0.5, min samples = 7. (**b**) DBSCAN eps = 1.5, min samples = 7. (**c**) DBSCAN eps = 1, min samples = 5.

In Figure 4, we present example influence of application of various DBSCAN parameters on detected objects bounding boxes on fragment of the dataset Kitti 39. Black dots are the reference vehicle path, red dots are the mapped path (they nearly overlap), blue boxes are vehicles positions, green dots are the point cloud of potential objects detected by the SLAM-OR approach, green boxes are estimations of objects’ positions estimated by our approach presented in Section 2.7.

**Figure 5:**
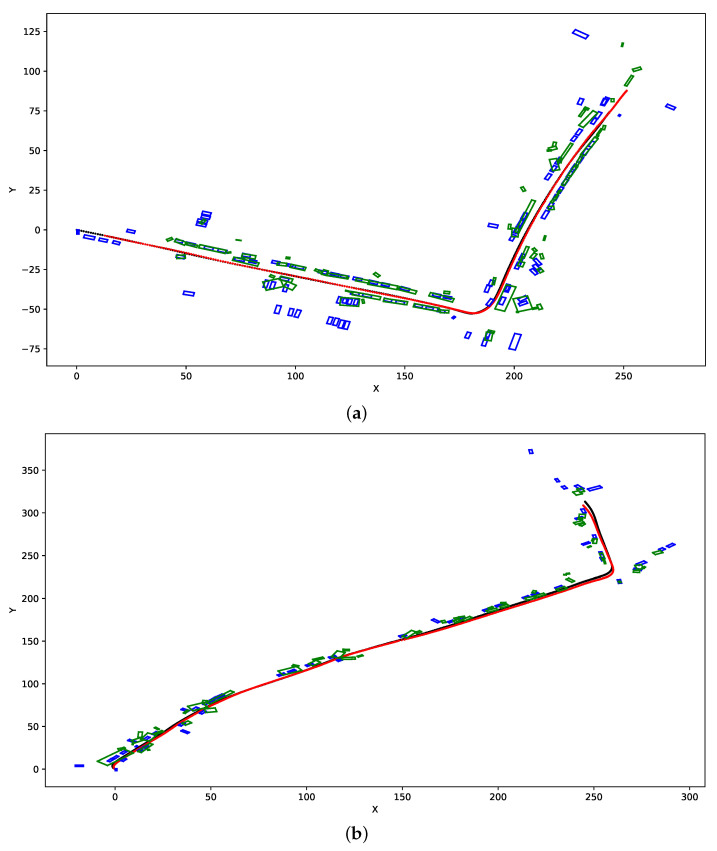
This figure presents example results of SLAM-OR algorithm with DBSCAN parameters: eps = 1 and min samples = 5. Black path and blue rectangles are reference data. Red line and green rectangles are results of the SLAM-OR. (**a**) SLAM and Bird’s Eye View mapping of Kitti 09 dataset. (**b**) SLAM and Bird’s Eye View mapping of Kitti 64 dataset.

In Figure 5, we present the example results of SLAM-OR bird’s eye maps that contain both estimated vehicles tracks and recognized object boxes. Those images present SLAM-OR results with DBSCAN parameters: eps = 1 and min samples = 5. The black path and blue rectangles are reference data. Red lines and green rectangles are results of the SLAM-OR. Both previously mentioned figures were generated for YOLO v4 network—MobileNet and RetinaNet generate very similar results.

## 4. Discussion

The proposed solution is constructed from the two key components, that is from simultaneous localization and mapping algorithm and objects detection and recognition algorithm that together enable spatial positioning of objects that are visible in video stream. Due to this, we have carefully evaluated both SLAM and OR algorithm in order to choose those solutions that obtain highest results among state-of-the-art approaches. As can be seen in Table 1 ORB SLAM is a stat- of-the-art vision-based algorithm that has very high translation and rotation accuracy in odometry/SLAM tests. As it was expected, stereo camera and LiDAR-based approaches have better precision; however, double camera and laser-based systems are far more expensive than single camera setup, which is an important issue, because proposed SLAM-OR approach does not relay on expensive hardware.

Among considered OR recognition algorithms the highest mAR has been obtained for MobileNet, YOLO v4 and RetinaNet (see Table 2). The training procedure for all considered networks is stable. As can be seen in Table 4, Table 5 and Table 6 and Figure 4 incorporation of the state-of-the-art SLAM and OR visual based approaches into single SLAM-OR framework that we have designed, enables to construct a reliable simultaneous localization, mapping and objects recognition framework. Both numerical and visual results of obtained paths and neighbouring objects bounding boxes confirm the quality of the final solution. The important issue is the proper choice of clustering parameters, which affects the number of detected objects and the volume of bounding boxes. Low eps causes underestimation of both objects sizes and volumes. If the value of eps is too high the algorithm has tendency to generate excessive bounding boxes and to join several separate objects together into single one. Small number of min samples parameter causes excessive detection of objects on recording while high value of this parameter might causes omitting existing objects. Furthermore, application of PCA-based bounding box size estimation seems to be accurate. Those observations are confirmed both in numerical and visual results. F1-score, which measures the accuracy of solution and Fowlkes–Mallows index that determine the similarity between two points distributions maximizes for YOLO v4 and MobileNet when eps = 1 and min samples = 5. In case of RetinaNet, maximal F1 and FM are obtained for eps = 1.25 and min samples = 5. There are not much difference between results obtained by three examined OR algorithms; however, highest FM score has been obtained for setup with RetinaNet, namely 0.43±0.09.

Example results presented in Figure 5 show, that SLAM-OR approach correctly calculated the track of the vehicle and assigns bounding boxes to objects that are situated on both sides of the path. The cause of most errors is lack of detection of objects that are relatively far away from vehicle path and that are partially occluded. Furthermore, some translation error in the bounding boxes positioning can be visible. That is caused by the fact that the SLAM-OR is based on relatively sparse point cloud which highly reduces calculation time of the whole solution due to really small number of points that are considered during clustering step. Due to the facts that points are taken directly from ORB SLAM algorithm it highly simplifies the further calculations by reducing amount of processed data. Of course the trade-off for this is an overall precision of bounding boxes detection. SLAM-OR has worse objects detection accuracy than state-of-the-art dense points LiDAR-based solutions; however, it has minimal data acquisition and computing hardware requirements to be operational. That makes our algorithm applicable in real time on portable low-powered devices like laptops and microcomputers. A dense point cloud generated from a LiDAR data maps only one side of objects that faces the laser sensor. In case of SLAM-OR a sparse point cloud seems to be spread across area that is occupied by the vehicle. This situation is visible for example in Figure 4b,c. This is caused by the fact that corner-based features that are detected by FAST algorithm might be situated not only in parts of objects that are close to the vehicle track but virtually in any object part that is visible from the camera perspective. Furthermore, data acquisition is performed three-dimensionally (not only in certain horizontal plane). Due to this PCA-based heuristic seems to operate on correct assumptions about data distribution; however, it might have tendencies to position object slightly closer to vehicle track. The detailed amount of this inaccuracy due to high variance of FAST points positioning seems not to be a systematic error, and due to this, we cannot compensate it easily.

## 5. Conclusions

The evaluation results presented in Section 3 and discussed in Section 4 prove that the proposed simultaneous localization, mapping and object recognition approaches using a visual sensor data is reliable algorithm that can successfully operate using a sparse point clouds. It has been successfully used in open environment benchmarks datasets obtaining satisfactory accuracy. As we already mentioned, SLAM-OR has worse objects detection accuracy than state-of-the-art dense points LiDAR-based solutions; however, it has minimal data acquisition and computing hardware requirements to be operational. We have designed SLAM-OR to be a first-choice algorithm in situations when there is a requirement of easy to deploy, fast and reliable algorithms for localization, mapping and neighbouring objects recognition for example in small-scale, low-power robotics. Algorithm can be easily adapted to recognize virtually any group of neighbour objects by well-established deep learning training framework. We have made all source codes of our method implementation available and we have utilized open data sets for evaluation, so our results can be easily reproduced and our algorithm can be quickly deployed.

## Figures and Tables

**Table 1:** Comparison of performance of selected odometry/SLAM algorithms on Kitti dataset. Columns Translation and Rotation present translation and rotation errors appropriately.

Method	Sensors	Translation	Rotation
VISO2-M+GP [85]	Single camera	7.46%	0.0245
ORB SLAM [69]	Single camera	6.23%	0.07
OV2SLAM [86]	Stereo camera	0.94%	0.0023
MULLS [87]	LiDAR	0.65%	0.0019

**Table 2:** This table presents value of loss function, data processing frequency (fps) and mean averaged precision (mAP) for networks after finishing the training.

Method	YOLO v3	YOLO v3 t	YOLO v4	YOLO v4 t	MobileNet	RetinaNet
Loss	12.01	10.28	4.44	9.29	–	–
fps	24.2	35.4	23.1	32.7	20.9	21.4
mAP	51.02	41.31	65.20	37.01	68.93	52.59

**Table 3:** Evaluation results of SLAM-OR (YOLO v4) algorithm depending of OPTICS parameters setup.

MIN	TPR	PPV	FNR	F1	FM
5	0.30 ± 0.10	0.20 ± 0.09	0.70 ± 0.10	0.23 ± 0.09	0.24 ± 0.09
10	0.27 ± 0.08	0.24 ± 0.13	0.73 ± 0.08	0.25 ± 0.11	0.25 ± 0.10
15	0.32 ± 0.08	0.26 ± 0.13	0.68 ± 0.08	0.27 ± 0.10	0.28 ± 0.09
20	0.34 ± 0.10	0.28 ± 0.13	0.66 ± 0.10	0.29 ± 0.11	0.30 ± 0.11
25	0.37 ± 0.10	0.26 ± 0.13	0.63 ± 0.1 0	0.29 ± 0.12	0.30 ± 0.11

**Table 4:** Evaluation results of SLAM-OR (YOLO v4) algorithm depending of DBSCAN parameters setup.

EPS	MIN	TPR	PPV	FNR	F1	FM
0.5	3	0.34 ± 0.15	0.35 ± 0.11	0.66 ± 0.15	0.33 ± 0.11	0.34 ± 0.11
0.5	5	0.27 ± 0.13	0.4 ± 0.14	0.73 ± 0.13	0.31 ± 0.12	0.33 ± 0.12
0.5	7	0.22 ± 0.11	0.44 ± 0.15	0.78 ± 0.11	0.28 ± 0.11	0.30 ± 0.11
0.75	3	0.44 ± 0.15	0.31 ± 0.10	0.56 ± 0.15	0.35 ± 0.10	0.36 ± 0.11
0.75	5	0.38 ± 0.14	0.35 ± 0.11	0.62 ± 0.14	0.36 ± 0.11	0.36 ± 0.11
0.75	7	0.32 ± 0.14	0.36 ± 0.12	0.68 ± 0.14	0.33 ± 0.11	0.33 ± 0.11
1	3	0.50 ± 0.15	0.27 ± 0.09	0.50 ± 0.15	0.34 ± 0.09	0.36 ± 0.09
1	5	0.46 ± 0.15	0.31 ± 0.10	0.54 ± 0.15	0.37 ± 0.10	0.38 ± 0.10
1	7	0.42 ± 0.15	0.34 ± 0.11	0.58 ± 0.15	0.37 ± 0.10	0.37 ± 0.11
1.25	3	0.54 ± 0.15	0.25 ± 0.08	0.46 ± 0.15	0.33 ± 0.09	0.36 ± 0.09
1.25	5	0.51 ± 0.15	0.28 ± 0.09	0.49 ± 0.15	0.35 ± 0.09	0.37 ± 0.10
1.25	7	0.48 ± 0.15	0.31 ± 0.09	0.52 ± 0.15	0.37 ± 0.10	0.38 ± 0.10
1.5	3	0.58 ± 0.15	0.23 ± 0.07	0.42 ± 0.15	0.32 ± 0.08	0.36 ± 0.08
1.5	5	0.56 ± 0.15	0.25 ± 0.08	0.44 ± 0.15	0.34 ± 0.09	0.37 ± 0.09
1.5	7	0.53 ± 0.15	0.28 ± 0.08	0.47 ± 0.15	0.36 ± 0.09	0.38 ± 0.09

**Table 5:** Evaluation results of SLAM-OR (MobileNet) algorithm depending of DBSCAN parameters setup.

EPS	MIN	TPR	PPV	FNR	F1	FM
0.5	3	0.34 ± 0.12	0.42 ± 0.12	0.66 ± 0.12	0.37 ± 0.10	0.37 ± 0.10
0.5	5	0.29 ± 0.10	0.50 ± 0.14	0.71 ± 0.10	0.36 ± 0.10	0.37 ± 0.10
0.5	7	0.25 ± 0.09	0.55 ± 0.13	0.75 ± 0.09	0.33 ± 0.10	0.36 ± 0.09
0.75	3	0.40 ± 0.12	0.38 ± 0.12	0.6 ± 0.12	0.38 ± 0.10	0.39 ± 0.10
0.75	5	0.37 ± 0.12	0.44 ± 0.13	0.63 ± 0.12	0.39 ± 0.10	0.40 ± 0.10
0.75	7	0.33 ± 0.10	0.49 ± 0.13	0.67 ± 0.10	0.39 ± 0.10	0.40 ± 0.10
1	3	0.45 ± 0.13	0.34 ± 0.11	0.55 ± 0.13	0.38 ± 0.10	0.39 ± 0.10
1	5	0.43 ± 0.12	0.39 ± 0.12	0.57 ± 0.12	0.41 ± 0.11	0.41 ± 0.11
1	7	0.40 ± 0.12	0.44 ± 0.13	0.60 ± 0.12	0.41 ± 0.11	0.41 ± 0.11
1.25	3	0.49 ± 0.14	0.31 ± 0.11	0.51 ± 0.14	0.37 ± 0.11	0.39 ± 0.11
1.25	5	0.47 ± 0.13	0.35 ± 0.12	0.53 ± 0.13	0.39 ± 0.11	0.40 ± 0.11
1.25	7	0.45 ± 0.13	0.39 ± 0.13	0.55 ± 0.13	0.41 ± 0.11	0.41 ± 0.11
1.5	3	0.52 ± 0.15	0.29 ± 0.11	0.48 ± 0.15	0.36 ± 0.11	0.38 ± 0.11
1.5	5	0.50 ± 0.14	0.32 ± 0.12	0.50 ± 0.14	0.38 ± 0.12	0.40 ± 0.12
1.5	7	0.49 ± 0.14	0.35 ± 0.13	0.51 ± 0.14	0.40 ± 0.12	0.41 ± 0.12

**Table 6:** Evaluation results of SLAM-OR (RetinaNet) algorithm depending of DBSCAN parameters setup.

EPS	MIN	TPR	PPV	FNR	F1	FM
0.5	3	0.37 ± 0.10	0.41 ± 0.13	0.63 ± 0.10	0.37 ± 0.09	0.38 ± 0.09
0.5	5	0.31 ± 0.08	0.47 ± 0.13	0.69 ± 0.08	0.36 ± 0.08	0.37 ± 0.08
0.5	7	0.26 ± 0.08	0.52 ± 0.14	0.74 ± 0.08	0.34 ± 0.07	0.36 ± 0.07
0.75	3	0.44 ± 0.10	0.37 ± 0.12	0.56 ± 0.10	0.39 ± 0.09	0.39 ± 0.09
0.75	5	0.39 ± 0.09	0.42 ± 0.13	0.61 ± 0.09	0.39 ± 0.09	0.39 ± 0.09
0.75	7	0.35 ± 0.09	0.44 ± 0.13	0.65 ± 0.09	0.38 ± 0.09	0.39 ± 0.09
1	3	0.50 ± 0.11	0.34 ± 0.12	0.50 ± 0.11	0.39 ± 0.10	0.40 ± 0.10
1	5	0.45 ± 0.10	0.38 ± 0.13	0.55 ± 0.10	0.40 ± 0.10	0.41 ± 0.09
1	7	0.42 ± 0.10	0.42 ± 0.14	0.58 ± 0.10	0.41 ± 0.09	0.42 ± 0.09
1.25	3	0.55 ± 0.11	0.31 ± 0.12	0.45 ± 0.11	0.38 ± 0.11	0.41 ± 0.10
1.25	5	0.52 ± 0.11	0.35 ± 0.13	0.48 ± 0.11	0.41 ± 0.10	0.43 ± 0.09
1.25	7	0.49 ± 0.11	0.39 ± 0.14	0.51 ± 0.11	0.41 ± 0.10	0.43 ± 0.10
1.5	3	0.60 ± 0.10	0.29 ± 0.11	0.40 ± 0.10	0.38 ± 0.11	0.41 ± 0.10
1.5	5	0.57 ± 0.10	0.32 ± 0.12	0.43 ± 0.10	0.39 ± 0.11	0.42 ± 0.10
1.5	7	0.54 ± 0.10	0.35 ± 0.13	0.46 ± 0.10	0.41 ± 0.11	0.42 ± 0.10

## Data Availability

Kitti dataset (real-world computer vision benchmarks in open environments): http://www.cvlibs.net/datasets/kitti/raw_data.php, accessed on 10 July 2021 [84]; Open Images v4 dataset (images and annotations): https://storage.googleapis.com/openimages/web/index.html, accessed on 10 July 2021; source codes of proposed method, its evaluation procedure and weights for DNN: https://github.com/PatrykMazurek/SLAM-OR.git, accessed on 10 July 2021.
